# Incidental Identification of an Intracranial Vertebral Artery Aneurysm Related to Unilateral Vertebral Artery Occlusion in a Patient With a Ruptured Internal Carotid-Posterior Communicating Artery Aneurysm: A Case Report

**DOI:** 10.7759/cureus.102088

**Published:** 2026-01-22

**Authors:** Kota Nakajima, Tamaki Kobayashi, Toshinari Kawasaki, Yoshihiko Ioroi, Yoshinori Maki, Motohiro Takayama

**Affiliations:** 1 Department of Neurosurgery, Graduate School of Medicine, Kyoto University, Kyoto, JPN; 2 Department of Neurosurgery, Kyoto Katsura Hospital, Kyoto, JPN; 3 Department of Neurosurgery, Hikone Chuo Hospital, Hikone, JPN

**Keywords:** anterior spinal artery aneurysm, intracranial, subarachnoid hemorrhage, unilateral, vertebral artery occlusion

## Abstract

An intracranial aneurysm located on the vertebral artery (VA) following unilateral VA occlusion is rare. Little has been described about this entity. An 85-year-old drowsy woman was transported to our hospital. A screening CT scan showed subarachnoid hemorrhage and secondary acute hydrocephalus. CT angiography revealed a right internal carotid-posterior communicating (IC-Pcom) artery aneurysm, an intracranial VA aneurysm, and left VA occlusion. The intracranial VA aneurysm was located on the lateral side of the left VA, near the origin of the anterior spinal artery. Following external ventricular drainage for acute hydrocephalus, the ruptured IC-Pcom aneurysm was obliterated with endovascular embolization. The intracranial VA aneurysm was not treated because of the complicated access route. The procedure was performed without any intraoperative complications. However, the patient died nine days after surgery because of global cerebral ischemia. Initial brain injury or rupture of the untreated intracranial VA aneurysm was suspected. We describe a rare case of an intracranial VA aneurysm that may have formed after unilateral VA occlusion.

## Introduction

The intracranial vertebral artery (VA) aneurysm is a relatively rare condition, accounting for 0.5-3.0% of intracranial aneurysms [1,2]. Morphologically, VA aneurysms can be divided into two groups: saccular and non-saccular. Non-saccular VA aneurysms are generally related to VA dissection and can be located on the VA trunk or a fenestrated limb. This type of VA aneurysm may coexist with stenotic lesions [3-5]. Meanwhile, saccular aneurysms can be located at vascular bifurcations and may result from hemodynamic stress [3-5]. To our knowledge, intracranial saccular VA aneurysms related to unilateral VA occlusion are scant in the literature. Here, we report a rare case of an intracranial saccular VA aneurysm possibly related to preceding unilateral VA occlusion.

## Case presentation

**Figure 1:**
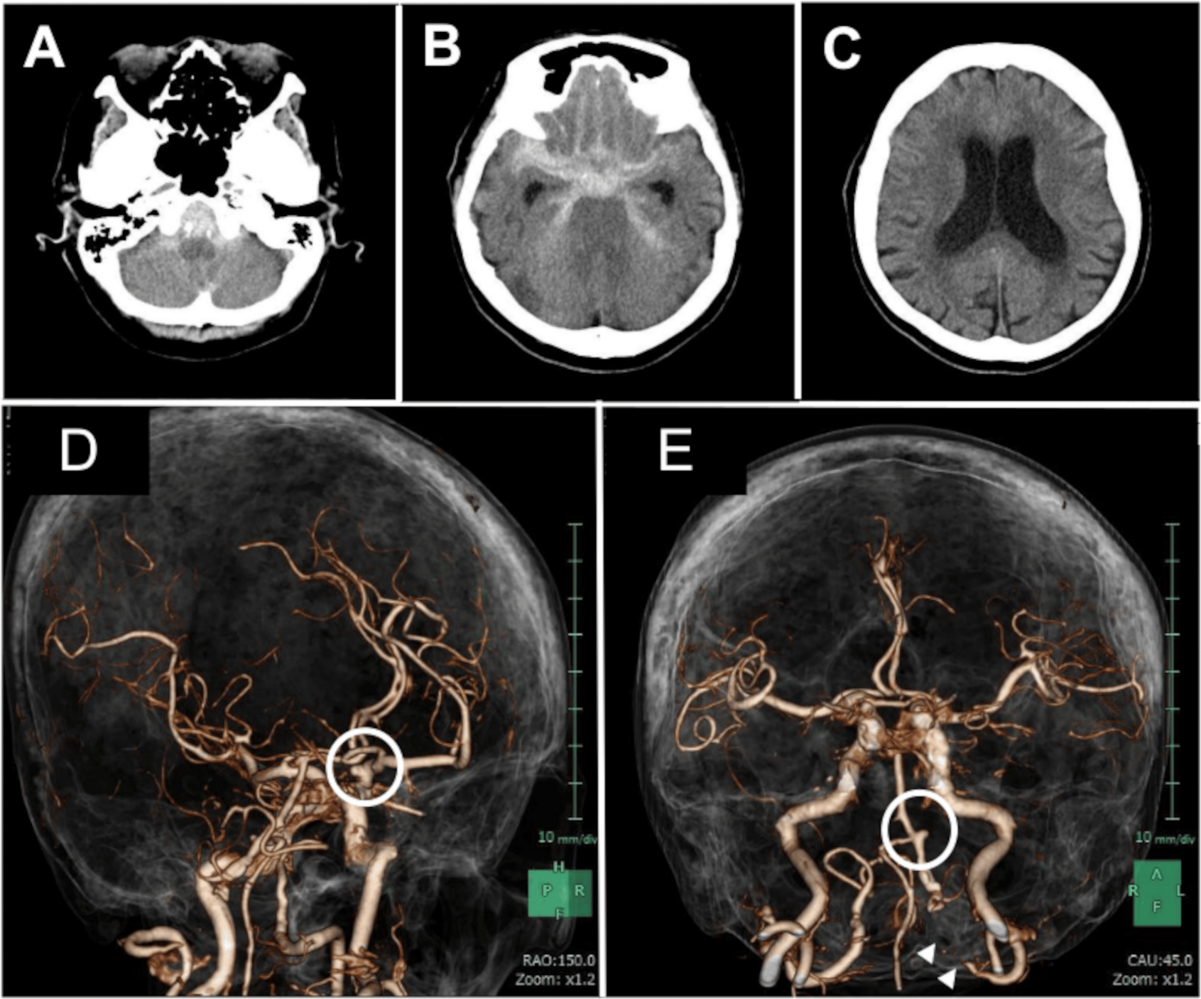
Preoperative computed tomography (CT) and CT angiography. (A, B) Subarachnoid hemorrhage (SAH) is identified in the posterior cranial fossa and basal cisterns, predominantly on the right side. (C) Concurrent acute hydrocephalus is observed. (D) A right internal carotid-posterior communicating artery aneurysm is observed (white circle). (E) A left saccular intracranial vertebral artery aneurysm is simultaneously identified on CT angiography (white circle). The left vertebral artery is occluded (white arrowheads).

An 85-year-old woman was transported to our ED. Her past medical history included hypertension, breast cancer, and right internal carotid (IC) artery and left VA dissection. The vascular dissections had been followed at another hospital for five years before the patient’s presentation. However, annual follow-up had ended because the patient did not develop any ischemic or hemorrhagic symptoms. Upon arrival, her neurological status was assessed as a Japan Coma Scale score of 10 and a Glasgow Coma Scale score of E3V2M5 (WFNS grade IV). Her systolic blood pressure was 190 mmHg. The bilateral pupil diameter was 3 mm, and the light reflex was prompt bilaterally. A head CT scan revealed subarachnoid hemorrhage (SAH) and concurrent acute hydrocephalus (Figure 1A-1C). Antihypertensive treatment was initiated, and the patient was sedated and intubated. On CT angiography, a right internal carotid-posterior communicating (IC-Pcom) artery aneurysm, a left saccular intracranial VA aneurysm, and left VA occlusion were simultaneously identified. The area around the left VA appeared to be perfused via the anterior spinal artery (ASA) from the right VA. Because the left VA aneurysm was oriented perpendicular to the VA pathway and parallel to the ASA, we hypothesized that it formed due to blood flow from the ASA (Figure 1D-1E).

**Figure 2:**
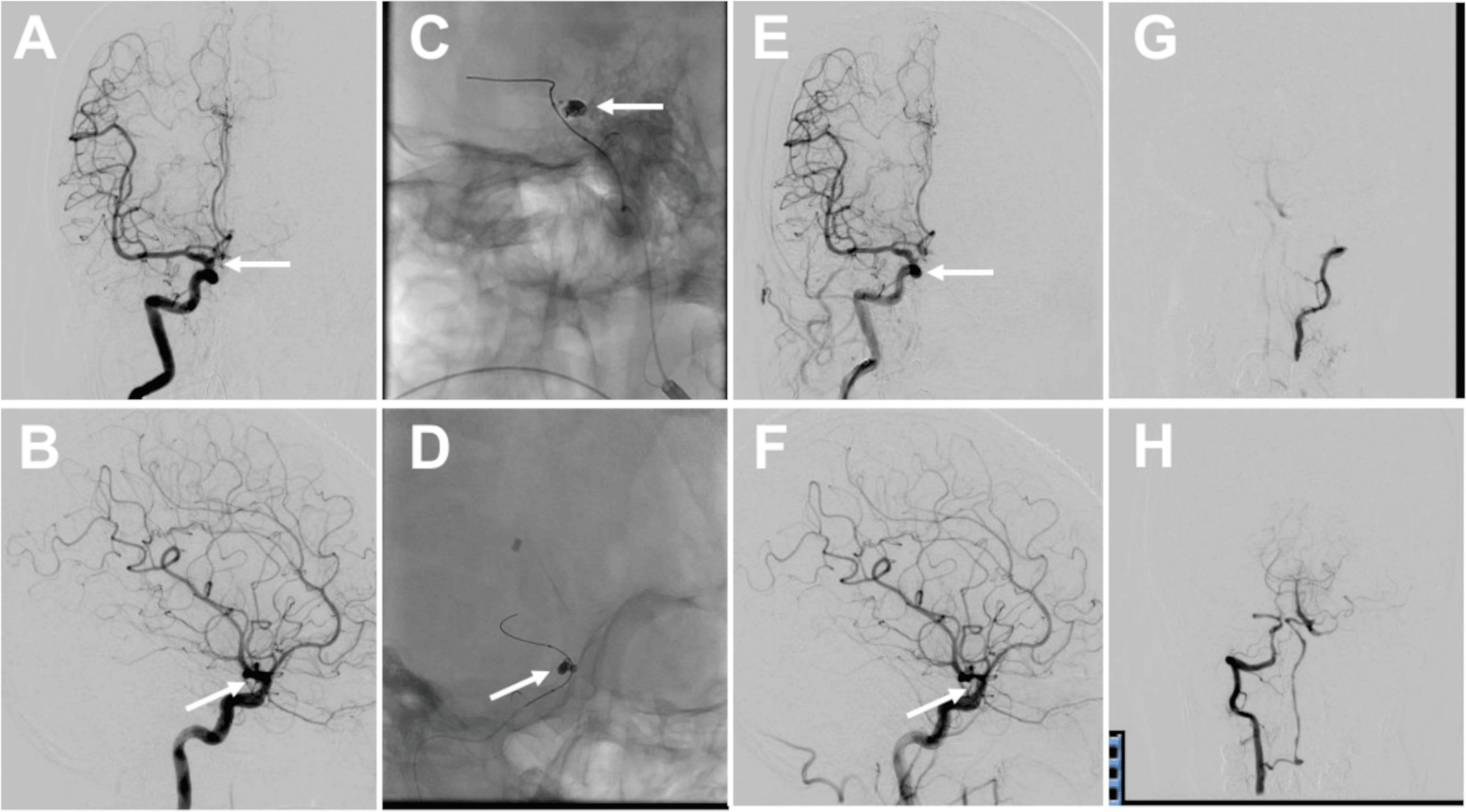
Intraprocedural findings. (A, B) The right internal carotid-posterior communicating (IC-Pcom) artery aneurysm (white arrows) was successfully embolized (A: anteroposterior projection; B: lateral projection). (C, D) After distal advancement of a microcatheter, the aneurysm neck was covered with a microballoon. The aneurysm was then successfully embolized (white arrows). (E, F) Final angiography confirmed successful embolization of the right IC-Pcom aneurysm (white arrows) (E: anteroposterior projection; F: lateral projection). (G) Left vertebral artery angiography demonstrated occlusion of the left vertebral artery. (H) Right vertebral artery angiography showed that the left posterior inferior cerebellar artery and the distal territory were supplied via the anterior spinal artery.

Based on the SAH distribution, we thought the IC-Pcom aneurysm could be the bleeding source. As the patient was elderly, we selected endovascular embolization of the IC-Pcom aneurysm and ventricular drainage to avoid the invasiveness of aneurysm clipping. Embolization of the IC-Pcom aneurysm was successful, and we did not identify any other vascular malformations (Figure 2).

**Figure 3:**
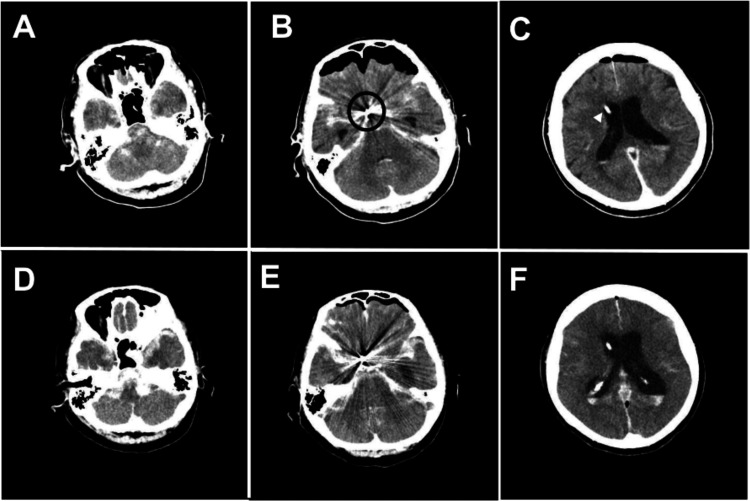
Postoperative computed tomography (CT) images. (A-C) After embolization of the right internal carotid-posterior communicating artery aneurysm (black circle), a drainage tube (white arrowhead) was placed in the anterior horn of the right lateral ventricle. Subarachnoid hemorrhage (SAH) in the posterior cranial fossa was not prominent. (D-F) CT images obtained one day after surgery showed diffuse cerebral ischemic changes. SAH in the posterior cranial fossa had increased.

A drainage tube was placed in the right anterior horn of the lateral ventricle (Figure 3A-3C). General anesthesia with propofol at 10 mL/h was continued after embolization, and daily aspirin 100 mg was introduced. After endovascular embolization, the bilateral pupil diameter was 2.5 mm, and the light reflex remained prompt bilaterally. However, the systolic blood pressure suddenly decreased to 60-70 mmHg the following day. This was followed by an increased volume of diluted urine, suggestive of diabetes insipidus. Repeat head CT showed global cerebral ischemia and increased SAH in the posterior cranial fossa (Figure 3D-3F).

The bilateral pupil diameter was 5 mm, and the light reflex disappeared. Although a cardiopulmonary event could also have occurred, brain damage from the initial SAH or rupture of the left saccular intracranial VA aneurysm was considered. As the patient’s family did not wish further treatment, intravenous infusion was continued for comfort measures only. The patient died nine days after surgery.

## Discussion

Here, we present a rare case of a saccular intracranial VA aneurysm concurrently identified with a ruptured right IC-Pcom artery aneurysm. A previous left VA occlusion accompanied the intracranial VA aneurysm. Although endovascular management of the ruptured aneurysm was achieved without difficulty, the patient died nine days later, possibly because of diffuse brain injury related to SAH, and possibly also because of rupture of the untreated intracranial saccular VA aneurysm.

Because intracranial VA aneurysms have a reported incidence of 0.5-3.0%, the incidence of saccular intracranial VA aneurysm, a subtype of VA aneurysm, is likely even lower [1,2]. In addition, reports concerning the coexistence of a saccular intracranial VA aneurysm and VA occlusion seem scant in the literature. Otaki Y et al. described an interesting case of a ruptured saccular intracranial aneurysm at the VA-ASA bifurcation for which endovascular embolization was performed [6]. They postulated that the intracranial ASA aneurysm formed because of hemodynamic stress. However, the intracranial aneurysm in their case was not related to VA occlusion; the right VA was only hypoplastic. In addition, the location of the ruptured aneurysm in their case was on the medial wall of the VA-ASA bifurcation [6], which differed from our case. Given the location of the VA aneurysm in our case, parallel to the ASA pathway and perpendicular to the VA pathway, the putative mechanism could have involved hemodynamic stress related to unilateral VA occlusion [7, 8]. As our patient had experienced left VA dissection, the left VA occlusion might have resulted from that previous event. In addition, there was no evidence of a congenital vascular anomaly in our case.

In this case, the VA aneurysm coexisted with a ruptured IC-Pcom aneurysm. Although endovascular embolization was successfully performed, the patient died nine days after surgery. Initial brain injury from SAH could have resulted in global cerebral ischemia and the unfavorable outcome. However, we also suspected that the untreated VA aneurysm could have ruptured. The surgical strategy for this VA aneurysm remains controversial. Open surgery on the VA in an elderly patient can be invasive. Endovascular embolization for the ruptured IC-Pcom and VA aneurysms in a single session can be an option; however, the access route via the right VA and ASA may be challenging. As the clinical follow-up for left VA occlusion at another hospital had been suspended, we did not have details regarding prior counseling or informed consent related to aneurysm management when the patient was transported to our hospital. However, with continuous clinical and radiological follow-up, preventive endovascular embolization for the intracranial VA aneurysm could have been considered. In this regard, regular follow-up should be warranted for similar cases.

## Conclusions

In this report, we describe a rare case of a saccular intracranial VA aneurysm that was incidentally and concurrently identified with a ruptured IC-Pcom aneurysm. The VA aneurysm may have developed secondary to preceding VA occlusion. Although the optimal management strategy in this setting remains controversial, given the invasiveness of open surgery and the challenging endovascular access, this case should be shared with clinicians.
